# A survey to evaluate animal methods bias experienced by India-based researchers in the peer review of manuscripts and grant applications

**DOI:** 10.1016/j.namjnl.2025.100042

**Published:** 2025-08-06

**Authors:** Surat Parvatam, Karishma Kaushik, Kasturi Mahadik, Goutami Nayak, Tejaswini Dhurde, Francesca Pistollato, Helder Constantino, Bipasha Gautam, Harshita Mittal, Catharine E. Krebs

**Affiliations:** a Humane World for Animals India, Hyderabad, India; b Indiabioscience, Bangalore, India; c Centre for Predictive Human Model Systems, Atal Incubation Centre-Centre for Cellular and Molecular Biology, Hyderabad, India; d Humane World for Animals Europe, Brussels, Belgium; e Physicians Committee for Responsible Medicine, Washington, DC, USA

**Keywords:** Nonanimal methods, Peer review bias, Grant application, Animal methods

## Abstract

While peer review during manuscript or grant application forms the cornerstone of scientific evaluation and assessment, this process is also prone to biases and confounding. Animal methods bias is a specific form of peer review bias where a preference for animal-based research methods or a lack of expertise in nonanimal-based methods undermines the quality and fairness of assessments of nonanimal studies. In this study, we conducted a survey to assess the challenges and experiences of researchers in India to publish peer-reviewed publications and apply for grants based on projects using nonanimal methods. A cross-sectional survey with 19 questions was completed by 186 respondents working in various biological fields in India. Decision logic was used to route respondents through the survey, which led to varying number of responses per question. Fifty-six per cent of respondents (39 out of 70) said they have been asked by manuscript reviewers to add animal experiments to their otherwise nonanimal-based studies. Respondents reported complying to 24 % of these requests on average. Respondents indicated the primary impacts of requests for additional experiments were publishing in lower impact factor journals and manuscript rejection. In addition, 57 % (47 of 83 respondents) felt that the lack of animal experiments in their grant proposal negatively influenced its evaluation. Respondents were also asked about key factors influencing their use of animal and nonanimal methods, which revealed that some perceived animal methods as necessary to validate nonanimal methods and as more reliable for mimicking biological complexity, while others perceived nonanimal methods as more physiologically relevant and practically advantageous. This survey provides preliminary evidence of animal methods bias experiences during publishing and funding peer review faced by Indian researchers.

## Highlights

• 56 % of India-based researchers surveyed (*n* = 39 out of 70) said they have been asked by manuscript reviewers to add animal experiments to their otherwise nonanimal-based studies.
• 57 % of respondents (*n* = 47 out of 83) felt that the lack of animal experiments in their grant proposal negatively influenced its evaluation.
• Respondents reported complying to one out of every four such of these requests on average (24%).
• 22 % of respondents (*n* = 16 out of 72) said they have used animal-based methods solely because they expected editor or reviewer requests for them.
• In terms of incentives to use animal-based methods, respondents indicated that they primarily used animal-based methods to validate in vitro findings (31 %) or mimic biological complexity (26 %).

## 1 Introduction

The scientific community evaluates the quality and merit of research via the peer review process; however, this process may be subject to reviewer bias that affects the objective evaluation of the research (Lee et al., 2013). During the peer review process, reviewers may ask authors to adjust analyses, reassess results, modify conclusions, or perform additional experiments, all of which can improve the quality of the study; but they can also introduce biases. This sort of peer review bias is characterized by the impartiality of the assessment, often caused by the dogma or pre-conceived notions of the reviewers (Haffar et al., 2019). Peer review bias can also impact the evaluation of grant applications and can be due to lack of training, inability to differentiate applications of similar strength, and weighing reputations and relationships (Sims Gould et al., 2025, Morgan et al., 2018).

In 2019, a perspective article was written by Donald Ingber titled, “Is it Time for Reviewer 3 to Request Human Organ Chip Experiments Instead of Animal Validation Studies?” which questioned the scientific and ethical reasoning for relying on animal testing for publication or grant funding (Ingber, 2020). In 2021, the journal editorial policies towards human-based versus animal-based methodologies to study research questions was deliberated upon during a panel discussion at the 11th World Congress on Alternatives and Animal Use in the Life Sciences (BioMed21 2025). Also referred to as animal methods bias, this is a form of peer review bias where a preference for animal-based research methods or a lack of expertise in nonanimal-based methods undermines the quality and fairness of assessments of nonanimal studies. For example, in a study that aimed to “establish long-term culture conditions of human airway epithelial organoids that contain all major cell populations and allow personalized human disease modelling” for cystic fibrosis, no animal experiments were initially used (Sachs, 2018). The corresponding author described the challenges of publishing this study without animal data (Triunfol, 2021), and eventually it was published after adding experiments that included transplanting the airway-organoids into immunocompromised mice (Sachs et al., 2019). The first systematic assessment of animal methods bias took place with a cross-sectional, online survey that collected the experiences and perceptions of researchers from around the world about manuscript peer review for nonanimal studies (Krebs et al., 2023). Survey findings indicated that 31 out of 68 respondents (46 %) had been asked by peer reviewers to add animal experimental data to their nonanimal study, just three of whom felt the request was justified. Twenty-one out of 68 respondents (31 %) said that they had carried out animal-based experiments for the sole purpose of anticipating reviewer requests for them—outside of the context of review, they did not think the experiments were necessary (Krebs et al., 2023).

In the past decade, several studies have analysed and documented the high sensitivity and specificity of human-relevant technologies, such as organ-on-chip and organoids towards specific contexts of use, such as liver toxicity (Ewart et al., 2022, Fäs et al., 2025). Another notable example includes the use of brain organoids which were instrumental to understand how Zika virus infection in pregnant women led to microcephaly in infants (Garcez et al., 2016). With the rising potential of these systems, the perception of animal models as the “gold standard” has also come under question (Ingber, 2020). As governments around the world are increasingly investing in nonanimal research approaches to overcome issues in translation and reduce and replace animal use, understanding the barriers to the broader use of these approaches is crucial. Animal methods bias can lead to delays in publication, publishing in lower-impact journals (Krebs et al., 2023), and grant rejections (Krebs et al., 2025) and thus constitutes one such barrier (Krebs and Herrmann, 2024).

India recently passed several policy and regulatory initiatives to advance the field of human-relevant and nonanimal research, including a white paper on the roadmap for developing alternatives to animals in experimentation (Swaminathan et al., 2019) and the New Drugs and Clinical Trials (Amendment) Rules, 2023, which allows the use of human biology-based test methods for testing the safety and efficacy of drugs (Ministry of Health and Family Welfare, 2023). However, both peer-reviewed publishing and funding (which could be impacted by peer review bias) are key drivers of research and innovation, and understanding the drivers and challenges of these parameters for India can guide the nation’s funding agencies, policymakers, and institutions to support human-relevant research more effectively. Thus, this study aimed to understand and document the peer review experiences and perceptions of researchers working in India when submitting manuscripts or grant applications based on projects using nonanimal methods and how this aligned or varied from the global trends. It is built on the previous survey described above, where geographically, subjects primarily conducted research in the US (32 %), followed by South Korea (15 %), Brazil (7 %), Italy (6 %), and UK (6 %) (Krebs et al., 2023). However, in addition to assessing researcher experiences with requests from peer reviewers for animal experiments, the survey probed researchers about their motivating factors for using animal and nonanimal methods, as well as their experiences with peer review when submitting grant proposals that included nonanimal data.

Investigations such as this one provide us with insights into the dimensions and characteristics of animal methods bias, including how it manifests in different countries and global regions where medical research funding, infrastructure, and laws and regulations related to the use of animals in research and testing differ. Understanding animal methods bias can help us determine better ways of mitigating its harmful effects on researchers, animals, and medical advancements (Kavanagh and Krebs, 2024).

## 2 Methods

### 2.1 Survey questions and logic

The CROSS checklist was followed in the reporting of this study (Supplementary Data S1) (Sharma et al., 2021). A cross-sectional survey was conducted using an online questionnaire via Qualtrics. The survey questions were based on the previous global survey to assess animal methods bias (Krebs et al., 2023), which was further adapted in conjunction with survey consultants at Faunalytics (https://www.faunalytics.org) and pilot tested with five subjects to refine the survey length and question clarity. Some of these questions were even further adapted to the India population of researchers and based on recent findings (unpublished data) (Supplementary Data S2). Participants were asked up to 19 questions. Logic routed subjects to the end of the survey if they did not currently work for an institution in India or if they had authored zero peer-reviewed publications (the first two questions after the consent form). Four questions followed about the subjects’ use of animal-based methods and nonanimal-based methods, respectively, in their research. Nine questions investigated subjects’ experiences with peer review, including two about grant proposal peer review. Lastly, four demographic questions were included. All questions were required except for three open-ended text response questions that asked participants to provide additional explanations about previous questions.

Decision logic was used to route respondents through the survey, which led to varying number of responses per question. For example, if they replied in affirmation to the question asking if they had been asked by a peer reviewer to add animal experiments to their study which otherwise had no animal data, then they were directed to a question asking how many times they had been asked to do this.

Responses were anonymized and the survey did not ask participants to provide identifiable information (Qualtrics did not record respondents’ IP Address, location data, and contact information). To further protect anonymity and confidentiality, qualitative and quantitative data were accessible to, and downloaded from the Qualtrics platform by a single researcher who checked that the data were fully anonymous before disseminating to team members (the co-authors of this manuscript). Incomplete survey responses were also recorded. Six respondents did not complete the survey: four failed to complete Q16-19, one failed to complete Q15-19, and one failed to complete Q13-19. Multiple submissions by the same respondent were prohibited. The survey was preceded by a consent form which included the information regarding the study purpose, study procedure, risks, inconveniences, and possible benefits, any associated costs of participation (none), confidentiality and data retention (data collected from this survey would be open access and publicly available at no cost), and possible contact persons in case of any questions or concerns.

In India, ethics committee approval is only required for research that involves clinical interventions, biomedical procedures, the use of human biological samples or identifiable personal health data. As the data collected by this survey does not involve any human biological samples or identifiable health data, it falls into the category of social science or behavioural research for which no ethical clearance is required in India. In addition, the survey collected no identifiable personal data, posed minimal or no risk to the participants, and took voluntary and informed consent at the start of the survey form. However, the authors still conducted an internal ethics assessment through the Internal Ethics Board of Humane World for Animals (formerly Internal Ethics Board of Humane Society International/India) before launching the survey, which also included external interest-holders. The survey protocol was reviewed and approved with the following Reference Code: HSII/IEB/002 – 2024.

### 2.2 Survey dissemination

The target population was researchers currently working in an Indian research institution who have published a nonanimal *in vitro* study. To target this population, a convenience sampling technique was used, in which the survey was disseminated via three India-based organisations: Humane World for Animals India, Centre for Predictive Human Models Systems (CPHMS) at the Atal Incubation Centre-Centre for Cellular and Molecular Biology (AIC-CCMB), and IndiaBioscience (IBS). The survey was disseminated both on social media platforms and email subscribers of these organisations. Six unique URL links were created to track responses coming in via the three organizations across two dissemination modes (social media and email).

The survey was disseminated between October 25, 2024, and January 15, 2025, and the close date was decided in advance. During this duration, emails were sent once a month by the three organisations amongst their respective network of researchers, and it was disseminated on social media once every 2 weeks. A total of 186 responses were received that were filtered for non-consent, respondents not working in India, zero publications, or missing responses to questions Q4/Q6, Q7, Q13, Q14 (Supplementary Data S2). A total of 100 remained after this filter criteria.

### 2.3 Survey analysis

The survey data was downloaded from Qualtrics on the date of closing of the survey and imported into Excel and R package for processing and analysis (Supplementary Data S3). Quantitative data analysis was performed by four of the co-authors. The responses to the three open-ended questions were independently coded by three reviewers who are also authors of this study (SP, KM, and TD), and SP took the lead in resolving any disagreements in coding through mutual discussions and performed the final compilation.

## 3 Results

### 3.1 Survey demographics

**Fig. 1:**
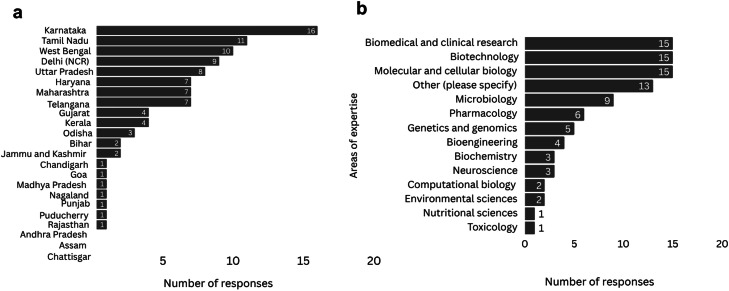
Survey demographics. The geographic distribution (a) and the area of expertise (b) of the survey respondents are shown.

Following the application of the filter criteria, the final sample included 100 respondents. Ninety-four respondents answered the question on the gender (as this was not a mandatory question), including 66 men (70 %), 26 women (30 %), and 2 non-binary persons (2 %; Q15). The respondents also ranged from across the different states in India (Q17), but were primarily from Karnataka (16 %), followed by Tamil Nadu (10 %), West Bengal (10 %), Delhi (10 %), Uttar Pradesh (9 %), Maharashtra (8 %), Telangana (8 %), and Haryana (7 %) (Fig. 1a). The research background of respondents (Q18) varied across several categories but primarily included molecular and cell biology (16 %), biomedical and clinical research (16 %), biotechnology (16 %), microbiology (8 %), pharmacology (6 %), genetics and genomics (5 %) (Fig. 1b). The respondents had around 47 published, peer reviewed publications on average (Q2) with an average time of 14 years since the highest degree (Q19). The number of responses from the three disseminating organisations were as follows: IBS (*n* = 59); Humane World (*n* = 19); CPHMS (*n* = 22).

### 3.2 Respondents’ use of animal- and nonanimal-based methods

Seventy-two per cent of survey respondents had used animal-based methods (Q3), and 70 % had used nonanimal-based methods (Q5). The survey also aimed to understand the key factors, including pressures or incentives, that have influenced respondents’ selection of animal- or nonanimal-based methods for their research via two open-ended questions (Q4, Q6).

There were 61 responses to the question about factors for animal-based methods (Q4). These responses were binned into eight qualitative categories (Supplementary Appendix S4). Primarily, respondents indicated that animal-based methods were required to validate the *in vitro* findings (31 %) or mimic biological complexity (26 %) (Table 1).

**Table 1:** Key factors, including pressures or incentives, influencing respondent’s selection of animal-based methods. The descriptive responses provided by the respondents in response to Q4 were binned to qualitative categories.

Category	*n* (%)	Description
Validate *in vitro* findings	19 (31 %)	Responses that indicated animal experiments were necessary to validate in vitro results
Mimic biological complexity	16 (26 %)	Responses that indicated that only in vivo models can mimic the complexities of human biology
Understand mechanisms of action	4 (7 %)	Responses that indicated that only in vivo models could provide mechanistic understanding of disease of interest
More reliable/valuable	4 (7 %)	Responses that indicated that only in vivo models were more reproducible/reliable/valuable
Perform proof of concept experiments	4 (7 %)	Responses that indicated in vivo models were used to conduct proof-of-concept experiments
Publication and peer review expectations	3 (5 %)	Responses that indicated in vivo models were used to obtain more impactful publication and acceptance by peer reviewers
Amenable to genetic manipulation	3 (5 %)	Responses that indicated in vivo models were used as it is easier to perform genetic manipulations
Practical advantages (monetary, reagents, existing ethics consent)	2 (3 %)	Responses that indicated in vivo models were used as they had practical advantages in terms of cost, etc.
Unavailability of human tissues/sample	2 (3 %)	Responses that indicated human samples or tissues were unavailable
No specific factor listed	4 (7 %)	NA

There were 62 responses to the question about key factors, including pressures or incentives, influencing respondents’ selection of nonanimal-based methods (Q6). These responses were binned into six qualitative categories (Supplementary Appendix S4). Primarily, respondents indicated that nonanimal-based methods could provide a relevant system to study human physiology (34 %), perform proof-of-concept experiments (13 %) and had practical advantages, such as monetary or ethical (21 %) (Table 2).

**Table 2:** Key factors, including pressures or incentives, influencing respondents’ selection nonanimal-based methods. The descriptive responses provided by the respondents in response to Q6 were binned to qualitative categories.

Category	*n* (%)	Description
Physiological relevance	21 (34 %)	Responses that indicated that in vitro models could recapitulate human physiology
Practical advantages (monetary, reagents, existing ethics consent)	13 (21 %)	Responses that indicated that in vitro models were easily available, cheaper, and did not involve obtaining ethical clearance
Perform proof of concept experiments	8 (13 %)	Responses that indicated that in vitro models were used for preliminary analysis, screening, establishing proof-of-concept
Understand mechanisms of action	7 (11 %)	Responses that indicated that in vitro models were used to understand underlying signalling pathways, biological mechanism, biochemical interactions etc.
Validate animal data	4 (6 %)	Responses that indicated that in vitro models were used to show conservation of mechanism between animals and humans
No specific factor listed	9 (15 %)	NA

### 3.3 Respondents’ experiences with publishing nonanimal-based studies

#### 3.3.1 Requests for animal experiments

**Fig. 2:**
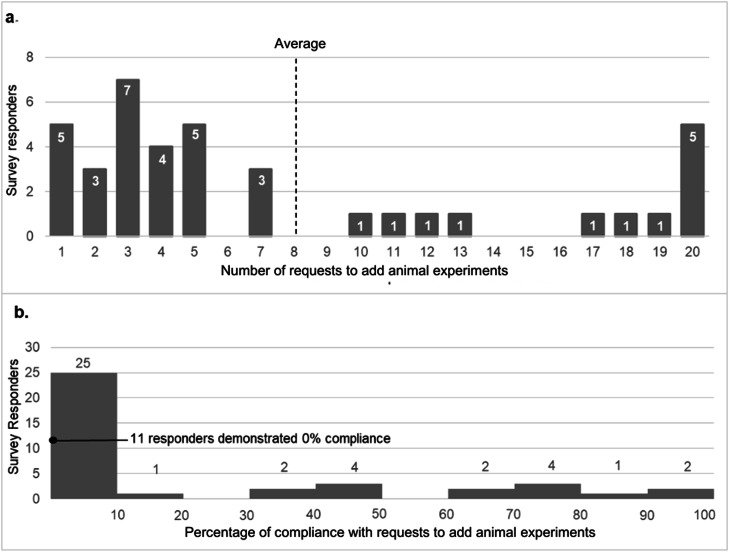
Number of requests for additional animal experiments experienced by the survey respondents and compliance rates. (a) The number of times a survey respondent had been asked by peer reviewers or editors for animal-based experiments to be added to their study, and (b) percentage of respondents’ compliance to these requests for additional animal experiments, displayed as a histogram.

Twenty-two percent of the respondents (*n* = 16 out of 72) said that they have used animal-based methods solely because they expected editor or reviewer requests for them (i.e., they did not think the experiments were necessary outside the review context; Q13). In response to the question if they had been asked by peer reviewers or editors to add animal experiments to a study which otherwise had no animal experiments (Q7), 56 % (39 out of 70 responses) replied in affirmative. The respondents who replied in affirmative were then directed to questions regarding number of times they had been asked (on a slider of 1-20, Q8) and the percentage of times they had complied with these requests (on a slider of 0 to 100, Q9). The researchers responded that they had been asked around eight times on an average to add animal-based experiments to be added to an otherwise nonanimal-based study (Fig. 2a). In addition, respondents complied on average to 24 % of requests (Fig. 2b). Specifically, out of 39 respondents (who had been asked to add animal experiments to their study), 11 (28 %) had never complied and 25 (64 %) had complied with fewer than 1 out of every ten requests (<10 %). Twenty-eight respondents (72 %) had complied at least once or more than once to requests for additional experiments (complied with more than 0 % of requests), and 37 respondents (95 %) had non-complied at least once or more than once to the requests for additional experiments (complied with less than 100 % of requests).

#### 3.3.2 Respondents’ reasons for compliance or non-compliance to requests for animal experiments

**Fig. 3:**
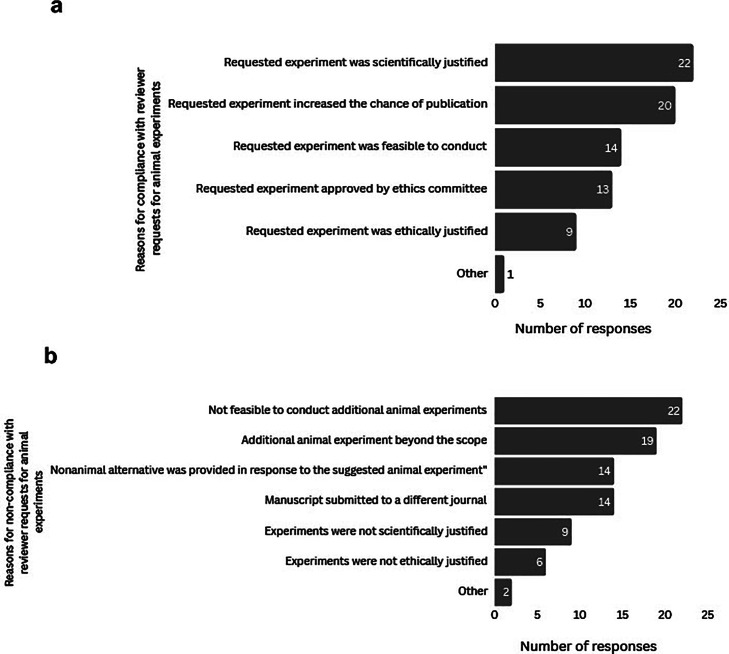
Respondents’ reasons for compliance or non-compliance to requests for animal experiments. Reasons stated by the respondents for compliance (a) and non-compliance (b) to requests for additional animal experiments.

We also probed to understand the reasons for compliance or non-compliance to these requests for additional animal experiments. The 28 respondents who had complied to requests for additional experiments (responded to % compliance as any number greater than 0 on the slider; Q9) indicated that the primary reasons for compliance were: the requested experiment was scientifically justified (*N* = 22), the requested experiment increased the chances of publication (*N* = 20), and the requested experiment was feasible to conduct (*N* = 14) (Fig. 3a). The 37 respondents who did not comply to the reviewer requests (responded to % compliance as anything less than 100 on the slider; Q11) indicated that the primary reasons for non-compliance were: it was not feasible to conduct the additional animal experiment (*N* = 22), the request for animal experiment was beyond the study’s scope (*N* = 19), and a nonanimal alternative was provided in response to the suggested animal experiment (*N* = 14) (Fig. 3b).

#### 3.3.3 Impact of reviewer requests for animal experiments

**Fig. 4:**
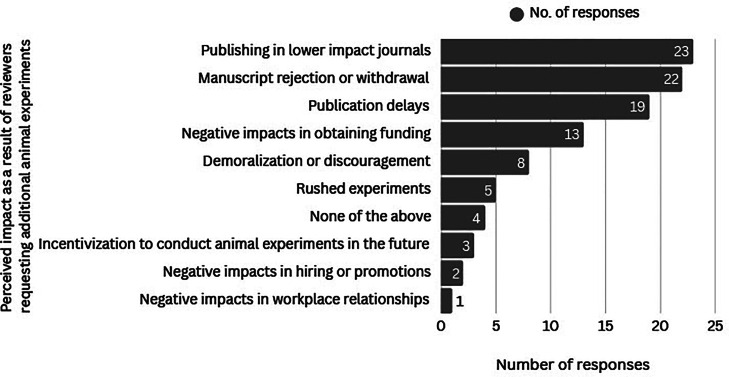
Impacts of reviewer requests for additional animal experiments. The incidence of various impacts perceived by the survey respondents as a result of reviewer requests for additional animal experiments (Q12).

We also sought to understand the possible impacts of requests for additional animal experiments on respondents. The 39 respondents who had experienced reviewer requests for additional animal experiments to their nonanimal data-based study reported experiencing several consequences as a result of these requests, including primarily publishing in lower impact journals (*n* = 23, 59 %), manuscript rejection or withdrawal (*n* = 22, 56 %), publication delays (*n* = 19, 49 %), and negative impacts in obtaining funding (*n* = 13, 33 %) (Q12; Fig. 4).

### 3.4 Impact of lack of animal experiments on grant applications

In addition to questions about experiences with peer reviewed publications, two questions also addressed the potential adverse impacts of lack of animal experiments on evaluations of grant applications. Of the 83 respondents who had submitted grants without animal experiments, 47 (57 %) felt that the lack of animal experiments in their grant proposal negatively influenced its evaluation (Q14). Another open question enquired about the perceptions of those 47 respondents about the impact of lack of animals on grant applications, asking respondents to explain what happened (Q15; Table 3). Thirty-eight responses were received for this open-ended question, which were binned into qualitative categories (Supplementary Appendix S4). Responses indicate that reviewers prefer animal-based methods due to both scientific and systemic pressures. These responses primarily ranged around perceptions, such as that animal experiments are required for validating in vitro experiments (42 %) and that proposals without animal experiments are considered to be lacking novelty or incomplete (37 %). Many researchers stated that the grant reviewers had expressed that validation of in vitro experiments using animal models was necessary.

**Table 3:** Respondents perceptions of how lack of animal experiments impacted their grant applications. The descriptive responses provided by the respondents on the perceived impact of lack of animal data on their grant applications (Q15) were binned into qualitative categories.

Category	*n* (%)	Description
Reviewers required animal-data based validation	16 (42 %)	Responses that indicated that animal data was necessary to validate in vitro data
Lack of animal experiments perceived to be lacking novelty or incomplete	14 (37 %)	Responses that indicated that in vitro models were insufficient to validate the results
Lack of understanding that animals may not be needed for that research	1 (3 %)	Responses that indicated that grant reviewer lacked understanding that animal model did not present an optimal model to investigate the research question
No relevant answer provided	7 (18 %)	NA

## 4 Discussion

In this study, we aimed to investigate animal methods bias—the bias towards animal-based methods—in publishing and grant applications, specifically experienced by researchers working in India. The findings provide preliminary evidence of challenges being faced by researchers in India in publishing nonanimal-based studies in peer-reviewed scientific journals and during the evaluation of grant applications and reveal systemic attitudes towards animal and nonanimal approaches.

The dissemination strategy aimed to collect the responses via organisational outreach through email and social media. For example, outreach was mainly conducted by IndiaBioscience (collecting 60 % of responses), which works with researchers across all sectors, and not primarily with researchers working with nonanimal methods. IndiaBioscience has one of the largest documented databases of researchers working in India in the area of life sciences, so the dissemination was expected to reach a large population of the target audience. However, the dissemination strategy did not reach Indian researchers who are not on any of the databases of the three organisations responsible for dissemination or who do not use social media platforms. In addition, outreach via Humane World for Animals could have biased the sample by recruiting participants with certain negative perspectives about animal use, although the dissemination specifically by Humane World contributed to 19 % of the survey responses. The convenience sampling method may have biased the sample, limiting generalisability, while also making it impossible to calculate response rate and non-response error, which could limit the reliability of survey results. However, the gender distribution of respondents are in line with a recent report indicating that women constitute 33 % of active researchers in India (Elsevier, 2024). A larger, more representative survey—capturing greater diversity across age and geographic regions within India—could help further substantiate and validate these findings. However, it should be considered that the number of researchers working on NAMs is still limited in India – one of the largest documented database of researchers working on microphysiological systems in India (MPS 2025) lists about 85 researchers as of July 2025 – limiting the maximum number of responses or scale that could be achieved for a survey like this.

With the shift in the use of human-relevant predictive systems globally both by the regulators and industry sector and the significant increase in the use of nonanimal methods in India (Parvatam et al., 2020), there is a need to understand the challenges researchers may face in publishing data or applying for funding with proposals based on these technologies, as such barriers could hinder their broader adoption. In a previous global survey of 68 researchers, 46 % of respondents (*N* = 31) indicated that they had been asked by reviewers to add animal data to their otherwise nonanimal-based experiments (Krebs et al., 2023). Of these 31 researchers, only 3 (9 %) complied with these requests and thought that it was justified. Confirming these previous findings and possibly indicating a larger issue in India, the results of the study herein shows that 56 % of respondents (39 out of 70) had been asked by manuscript reviewers to include animal data in their otherwise nonanimal-based studies. In addition, in the global survey, 31 % (*N* = 21/68) of survey respondents mentioned that they have performed animal-based experiments for the sole purpose of anticipating reviewer requests for them, whereas in the India survey, this percentage was 22 % (16/72).

On average, respondents complied with only one out of every 4 requests to add animal experiments (Fig. 2b), and the reasons for non-compliance included lack of feasibility, the use of a nonanimal method in place of animal experiments, and lack of scientific justification, among others. This non-compliance was further perceived to translate to manuscript rejection, publishing in lower impact journals, and negative impact on the possibility to obtain funding, among other outcomes (Fig. 4). Consequences related to impact factor, rejections, and delays were also identified in the previous global survey, with one respondent stating that: “The journals that wanted animal experiments were journals with higher impact factors… There is no incentive to not do animal experiments” (Krebs et al., 2023). Such perceptions can lead to hindrances in wider adoption and usage of these systems. The two open-ended questions that prompted respondents to describe the key factors for using animal- or nonanimal-based methods further highlighted animal methods bias (Table 1, Table 2 and Supplementary Appendix S4). Many researchers commented that animal-based methods are valuable, reliable, and provide more physiologically relevant responses, despite the presence of evidence to the contrary. Animal-based methods were perceived as critical for manuscript acceptance in high-impact journals, for career advancement, and for meeting ethical standards in preclinical research. Recently, several studies have highlighted the reproducibility crisis in preclinical research that currently primarily relies on animals (Baker, 2016, Hunter, 2017), and in specific contexts of use, nonanimal methods have been shown to be more predictive than animals. In addition, it was predominantly perceived that animal-based methods are required for validating nonanimal experiments where 31 % (*N* = 19/61) of respondents said they used animal-based methods for this reason (Table 1). Animal experiments have been traditionally considered the gold standard for research; however, the predictivity of animals to human patient outcomes has been questioned in the recent decade (Ingber, 2020). The lack of translation of preclinical animal data to humans has been considered as one of the reasons for high failure rate during clinical trials, costing pharmaceutical companies billions of dollars and years of lost time (Hartung, 2024). Overall, provided comments suggest a deep entrenchment of animal methods in both scientific practice and publication culture, with a prevailing belief that meaningful or impactful experimental biology cannot yet be achieved without them.

On the other hand, regarding the use of nonanimal methods, the responses suggest a growing recognition of the scientific and ethical strengths of nonanimal approaches, particularly when human relevance and mechanistic insights are prioritized. However, several survey respondents stated they use nonanimal methods during the “proof-of-concept” stage, implying that, in some cases, nonanimal methods are still viewed primarily for initial studies and complementary to, rather than complete replacements for (or alternatives to), traditional *in vivo* studies.

This was also mirrored in the responses regarding researchers’ perception of the impacts of lack of animal experiments on grant applications (Table 3). Among comments provided, some respondents highlighted that reviewers saw animal experiments as required for validation, or that proposals lacking animal experiments lacked novelty or were judged as incomplete (Supplementary Appendix S4), both of which could reasonably reduce the likelihood of securing funding. These responses indicate a systemic bias toward animal experiments and lack of awareness regarding the recent advancements in the use of nonanimal new approach methods (NAMs) for preclinical research.

A recent survey study gathering industry-wide feedback regarding nonanimal methods usage showed that 54 % of responding pharmaceutical companies used a nonanimal method even when a large animal species was available, and 91 % considered using nonanimal methods to replace traditional large animals in future studies (Shenton et al., 2025). In addition, companies have used NAMs to support all stages of drug development, including preclinical development (prior to regulatory submission for first-in-human [FIH] trials) (42 % of respondents), FIH studies (21 % of respondents), or registration (21 % of respondents). Recognising the limitations of animal models, both the US Food and Drug Administration (FDA) (under the FDA Modernization Act 2.0 (Sen and Paul, 2022) and 3.0 (Rep. Carter, 2025)) and the Ministry of Health and Family Welfare, Government of India (Ministry of Health and Family Welfare, 2023) authorised the use of alternatives to animal testing, including cell-based assays and computer models to test the safety and efficacy of a drug. The US FDA also recently released a roadmap towards reducing animal studies for preclinical safety assessment that encourages the sponsors to submit nonanimal data, and provided assurance of regulatory relief (e.g., fewer animal study replicates) if such data was submitted (U.S. Food and Drug Administration, 2025).

In the light of these global regulatory and industry changes towards the use of human-relevant predictive models rather than animal models, there is a need to understand systemic biases that may act as an impediment towards greater use, development, and adoption of nonanimal models so they may be mitigated. In the previously published report of the global survey, some of the causal factors underlying animal methods bias in publishing were discussed, including preferences for animal methods or lack of awareness of the existing nonanimal methodologies to study different contexts of use in biomedical and translational research (Krebs et al., 2023). While further investigation would be required to understand the causal factors in the context of the Indian research community, the predominant perception that emerged from the descriptive responses indicated that without animal data, studies were perceived as being incomplete, and lacking in novelty or value. This suggests that reviewers may be unaware of the value of nonanimal methods or may lack expertise to properly evaluate them, as also highlighted in the global survey.

## 5 Conclusions

These findings provide preliminary evidence of the challenges being faced by researchers in India to publish or obtain grant funding based on research carried out using nonanimal-based methods, where 56 % of respondents (39 out of 70) said that had been asked by manuscript reviewers to add animal experiments to their otherwise nonanimal-based studies, leading to manuscript rejection and publishing in lower impact journals. In addition, 57 % had felt that the lack of animal experiments in their grant proposal negatively influenced its evaluation. Twenty-two per cent of respondents have performed animal-based experiments for the sole purpose of anticipating reviewer requests for them. The open-ended questions on the reasons for the use of animal or nonanimal methods also indicated systemic beliefs, including that animal methods were deemed more valuable or reliable and necessary to validate in vitro findings.

Strategies have been proposed to mitigate animal methods bias, addressed to both researchers (such as providing robust justifications of model choice, validating findings in confirmatory experiments based on nonanimal models, and appropriately framing findings and conclusions (Kavanagh and Krebs, 2024, Krebs et al., 2023)) and funders (such as increasing investment in nonanimal research, training, and infrastructure, educating the scientific community about the value of nonanimal approaches, and ensuring review groups have appropriate nonanimal expertise (Kavanagh and Krebs, 2024)). With the global technology and regulatory shift towards the use of human-based research technologies, including in India, where it is being recognized that these technologies can expand the toolbox for researchers to answer complex biomedical research questions, such studies can help funding agencies, policymakers, and institutions identify challenges and drivers to enable this change.

## Funding

This work was supported in part by Lush Handmade Cosmetics Ltd. [Advancing Animal Free Research Models] to CEK.

## CRediT authorship contribution statement

**Surat Parvatam:** Writing – original draft, Visualization, Validation, Supervision, Project administration, Methodology, Investigation, Formal analysis, Data curation, Conceptualization. **Karishma Kaushik:** Writing – review & editing, Resources, Methodology. **Kasturi Mahadik:** Writing – review & editing, Visualization, Formal analysis. **Goutami Nayak:** Writing – review & editing, Methodology. **Tejaswini Dhurde:** Visualization, Methodology. **Francesca Pistollato:** Writing – review & editing. **Helder Constantino:** Writing – review & editing, Conceptualization. **Bipasha Gautam:** Resources, Methodology. **Harshita Mittal:** Writing – review & editing. **Catharine E. Krebs:** Writing – review & editing, Software, Project administration, Methodology, Funding acquisition, Formal analysis, Data curation, Conceptualization.

## Declaration of competing interest

The authors declare the following financial interests/personal relationships which may be considered as potential competing interests:

One of the co-authors, Francesca Pistollato is on the editorial board of the NAM Journal, but she had no involvement in the peer review of the paper and had no access to its peer review. Full responsibility for the editorial process for this article was delegated to another journal editor. If there are other authors, they declare that they have no known competing financial interests or personal relationships that could have appeared to influence the work reported in this paper.

## Data Availability

The data that support the findings of this study are available in Supplimentary Data S3.

## Appendix Supplementary materials

application 1 Image, application 1

application 2 Image, application 2

application 3 Image, application 3

application 4 Image, application 4
