# Supplementary material for: A survey to evaluate animal methods bias experienced by India-based researchers in the peer review of manuscripts and grant applications
Source: NAM J. 2025 Aug 6;1:100042. doi: 10.1016/j.namjnl.2025.100042 (PMC13288646; doi:10.1016/j.namjnl.2025.100042)
Supplement: application 2 [file mmc2.pdf]

Experiences with reviewer requests for animal experiments in India

Start of Block: Consent

Consent
Informed Consent Form
Sponsor: Humane World for Animals India/IndiaBioscience/ Centre for Predictive Human Model Systems, Atal Incubation Centre-Centre for Cellular and Molecular Biology
Study Title: "Experiences with reviewer requests for animal experiments in India"
Principal Investigator: Surat Parvatam, PhD
Telephone: +91-9892396861
Address: Humane World for Animals India
8-2-332/5/1, Rd Number 3 Banjara Hills, Hyderabad, Telangana 500034

You are invited to take part in this research project because you are at least 18 years of age, are currently working in an Indian research institution, and have published a nonanimal *in vitro* study in the last two years. Your participation in this study is voluntary, and a decision to withdraw or not to participate will not be used against you in any way and will not result in any penalty or loss of benefits to which you are otherwise entitled. The investigator or the sponsor can stop your participation at any time without your consent for the following reasons:

- If you fail to follow directions for participating in the study;
- If it is discovered that you do not meet the study requirements;
- If the study is canceled; or
- For administrative reasons.

Study Purpose: To understand the prevalence of peer reviewer requests for animal-based experiments to be added to studies performed without the use of animals and authors' perceptions of these requests in India. About 385 subjects will participate in this study.

Study Procedure: If you agree to take part in this survey, we will ask you to answer a few demographic questions about yourself, questions about your use of research animals and other methods, as well as questions about your experiences and perceptions during manuscript submission when peer reviewers request animal-based experiments to be added to studies performed without the use of animals. Responses to all questions are required, except open-ended questions. The survey is estimated to take about 5 minutes total.

Risks, Inconveniences, and Possible Benefits: We anticipate minimal risks to participating in this study, no greater than you would encounter in your everyday life. There may be risks that

are unknown. You may not receive any direct benefit from your participation in this study, but your participation will improve researchers' understanding of the subject.
Alternatives: This research study is for research purposes only. The only alternative is to not participate in this study.
Costs: There will be no charge to you for your participation in this study.
Withdrawing from the Study: Because this study is completely anonymous, if you withdraw your consent during the course of the study, we will be unable to identify and remove any data you provided before withdrawal.
Confidentiality and Data Retention: You are not required to provide any identifying information to participate in this study. After the study is completed and identifying information is removed, the data from the study will be made open access (publicly available at no cost) so that other researchers can use it. The results of this research study may be presented at meetings or in publications but your identity will not be disclosed. While every effort will be made to protect the confidentiality of your information, absolute confidentiality cannot be guaranteed.
Whom to Contact About This Study: During the study, if you have questions, concerns or complaints about the study such as: • Your responsibilities as a research subject; • Eligibility to participate in the study; • The investigator's or study site's decision to withdraw you from participation;
Please contact the investigator at the telephone number listed on the first page of this consent document. An institutional ethics board is an independent committee established to help protect the rights of research subjects. If you have any questions about your rights as a research subject, contact

By **mail**: Institutional Ethics Board, Humane World for Animals India, 8-2-332/5/1, Road No. 3, Banjara Hills, Hyderabad, Telangana 500034 or **telephone**: 040 23552601/02 or **email**: india@hsi.org. Please reference the following code when contacting the institutional ethics board: HSII/IEB/002 – 2024.
Statement of Consent: If you understand the above information and voluntarily agree to participate in this study, please indicate your consent below. If you have questions, you can contact the investigator before continuing.

○ I consent to participate and understand the information provided to me above

○ I DO NOT consent (this will eject you from the survey)

Definitions **PLEASE READ CAREFULLY - Survey term definitions** You will be asked questions about animal-based and nonanimal-based experiments. Please read the following definitions of these terms carefully. **Animal-based experiment**: An experiment performed in a living non-human animal or in a non-human animal-derived organ, tissue, or other biological product, e.g., an animal *in vivo* model or animal cell-derived *in vitro* model. **Nonanimal-based experiment**: An experiment performed in a living human, in a human-derived organ, tissue, or

cell-based *in vitro* model, even if it uses animal-based materials such as antibodies or culture sera, or *in silico*, e.g., deep learning or artificial intelligence.

End of Block: Instructions

Start of Block: Country filter

Q1 Do you **currently** work for an institution in India?

○ Yes

○ No

Q2 On approximately how many peer-reviewed publications are you listed as author? (If greater than 300, leave the slider at 300. If zero, move the slider to the right and then back to zero.)

	0 30 60 90 120 150 180 210 240 270 300

Q3 In ALL your studies EVER, including non-published work, have you used **animal-based** methods (experiments performed in a living non-human animal or in a non-human animal-derived organ, tissue, or other biological product)?

○ Yes

○ No

Q4 What key factors, including pressures or incentives, have influenced your selection of **animal-based** methods? Please explain in one or two sentences.

____

Q5 In ALL your studies EVER, including non-published work, have you used **nonanimal-based** methods (experiments performed in a living human, in a human-derived organ, tissue, or cell-based *in vitro* model)?

○ Yes

○ No

Q6 What key factors, including pressures or incentives, have influenced your selection of **nonanimal-based** methods? Please explain in one or two sentences.

____

Q7 During a manuscript submission process, have you EVER been asked by peer reviewers or editors for **animal-based** experiments to be added to a study of yours that otherwise had **no** animal-based experiments?

○ Yes

○ No

Q8 Approximately how many times have you been asked by peer reviewers or editors for **animal-based** experiments to be added to a study of yours that otherwise had **no** animal-based experiments? (If greater than 20, leave the slider at 20.)

1 3 5 7 9 11 12 14 16 18 20

Q9 Approximately what percentage of these requests for additional animal experiments did you comply with? (If zero, move the slider to the right and then back to zero.)

0 10 20 30 40 50 60 70 80 90 100

Q10 For instances when you **complied** with the requests for additional animal experiments, why did you do so? Select all that apply.

- ☐ The additional animal experiments were scientifically justified.
- ☐ The additional animal experiments were ethically justified.
- ☐ The additional animal experiments increased the chance of publication.
- ☐ It was feasible to conduct the additional animal experiments with time, resources, facilities, expertise, and/or collaborators.
- ☐ The additional animal experiments were approved by the appropriate ethics committee.
- ☐ Other (please specify): ____

Q11 For instances when you **didn't comply** with the requests for additional animal experiments, why didn't you do so? Select all that apply.

- ☐ The additional animal experiments were not scientifically justified.
- ☐ The additional animal experiments were not ethically justified.
- ☐ A nonanimal alternative was provided in response to the suggested animal experiments.
- ☐ The manuscript was submitted to a different journal.
- ☐ It was not feasible to conduct the additional animal experiments with time, resources, facilities, expertise, and/or collaborators.
- ☐ The additional animal experiments would have been beyond the study's scope.
- ☐ Other (please specify): ____

Q12 Have you EVER experienced any of the following as a result of reviewers requesting additional animal experiments? Select all that apply.

- ☐ Publication delays
- ☐ Manuscript rejection or withdrawal
- ☐ Publishing in lower impact journals
- ☐ Demoralization or discouragement
- ☐ Incentivization to conduct animal experiments in the future
- ☐ Negative impacts in obtaining funding
- ☐ Negative impacts in hiring or promotions
- ☐ Negative impacts in workplace relationships
- ☐ Rushed experiments
- ☐ Other (please specify): ____
- ☐ ⊗None of the above

Q13 Have you EVER used animal-based methods **solely** because you expected peer reviewer or editor requests for them (i.e., you did not think the experiments were necessary outside of the review context?)

○ Yes

○ No

Q14 Have you EVER felt that the lack of animal experiments in your **grant proposal** negatively influenced its evaluation? If you're not sure, give your best guess.

○ Yes

○ No

○ Not applicable (I've never submitted a grant without animal experiments)

Q15 If yes (if you've felt that the lack of animal experiments in your **grant proposal** negatively influenced its evaluation), can you please explain what happened in one or two sentences?

____

Q16 What is your gender?

- ○ Man
- ○ Woman
- ○ Non-binary
- ○ I do not see myself represented in the above options. My gender is:
____
- ○ I prefer not to answer.

Q17 In which state or union territory do you **primarily** work?

- ○ Andaman and Nicobar Islands
- ○ Andhra Pradesh
- ○ Arunachal Pradesh
- ○ Assam
- ○ Bihar
- ○ Chandigarh
- ○ Chhattisgarh
- ○ Dadra and Nagar Haveli and Daman and Diu
- ○ Delhi (National Capital Territory)
- ○ Goa
- ○ Gujarat
- ○ Haryana
- ○ Himachal Pradesh
- ○ Jharkhand

- ◯ Jammu and Kashmir
- ◯ Karmataka
- ◯ Kerala
- ◯ Ladakh
- ◯ Lakshadweep
- ◯ Madhya Pradesh
- ◯ Maharashtra
- ◯ Manipur
- ◯ Meghalaya
- ◯ Mizoram
- ◯ Nagaland
- ◯ Odisha
- ◯ Punjab
- ◯ Puducherry
- ◯ Rajasthan
- ◯ Sikkim
- ◯ Tamil Nadu
- ◯ Telangana
- ◯ Tripura
- ◯ Uttar Pradesh
- ◯ Uttarakhand

- West Bengal

Q18 What best describes your **primary** field of research?

- Biochemistry
- Bioengineering
- Biomedical and clinical research
- Biophysics
- Biotechnology
- Computational biology
- Dentistry
- Environmental sciences
- Genetics and genomics
- Microbiology
- Molecular and cellular biology
- Neuroscience
- Nutritional sciences
- Pharmacology
- Physiology
- Social sciences
- Systems biology
- Toxicology
- Other (please specify): ____

Q19 Approximately how many years has it been since you received your highest earned degree? If greater than 50, leave the slider at 50.

	0 5 10 15 20 25 30 35 40 45 50
